# Anticipation and sequential demands influence on-field change-of-direction kinematics related to ACL injury risk

**DOI:** 10.3389/fspor.2026.1659044

**Published:** 2026-02-23

**Authors:** Mareike Kühne, Christian Sanin, Maurice Mohr

**Affiliations:** Department of Sport Science, University of Innsbruck, Innsbruck, Austria

**Keywords:** biomechanics, cutting, IMUs, knee injury, neurocognition, sidestepping, unplanned, wearable sensors

## Abstract

**Introduction:**

Anterior Cruciate Ligament (ACL) injuries are a significant concern in multidirectional sports, often occurring during change-of-direction (COD) maneuvers under high biomechanical loads. While laboratory-based studies have provided valuable insights into ACL injury mechanisms, they often fail to replicate the complexity of real-world scenarios. This cross-sectional study aimed to investigate the kinematic patterns and performance of COD maneuvers in ecologically valid settings, incorporating on-field testing with reduced constraints, multiple angles, and both preplanned and unplanned conditions. Additionally, the study examined how kinematics evolve during a sequence of CODs, simulating game-like scenarios.

**Methods:**

Twenty male soccer players performed COD sequences on artificial turf, with joint and segment kinematics captured using wearable inertial measurement units (IMUs).

**Results:**

Results revealed that limiting movement planning did alter COD movement executions and elicited a mix of protective and risk-associated movement adaptations. Unplanned CODs exhibited less favorable trunk and pelvis alignment in regard to ACL injury risk, but they also showed safer sagittal knee and frontal hip kinematics, however, at the cost of performance. Notably, kinematic patterns deteriorated in the last COD of an unplanned sequence, with participants displaying characteristics associated with elevated injury risk: more extended knees, higher hip abduction at sharper angles, and misaligned trunk rotation.

**Discussion:**

These findings suggest that the combined cognitive and physical demands of sequential CODs constrain motor planning, leading to riskier biomechanical patterns. This study underscores the importance of incorporating realistic, game-like conditions in ACL injury research to better understand the interplay between performance and injury risk. The results highlight the need for injury prevention programs to address the cognitive and physical demands of unplanned, sequential CODs, offering a more comprehensive approach to mitigating ACL injury risk in multidirectional sports.

## 1 Introduction

Anterior Cruciate Ligament (ACL) ruptures are among the most debilitating musculoskeletal injuries in sports, entailing long healing and rehabilitation processes and possible long-term functional impairments or reinjuries (1). ACL injuries frequently occur during change-of-direction (COD) movements and without opponent contact (2–4). Therefore, they remain a persistent concern and topic of interest in multidirectional sports such as soccer (5), where players perform about 100 up to 300 CODs per game (6, 7). The biomechanics of CODs, specifically the multiplanar knee joint loads and associated kinematic postures during the COD ground contact phase, have gained attention in sports science in order to better understand ACL injury mechanisms and risk factors for prevention purposes (8, 9). COD maneuvers typically involve decelerating the whole-body center-of-mass (COM), followed by a reorientation and reacceleration into a new movement direction within a few steps including a prominent main foot plant, a sidestep (10). At or shortly after (∼50 ms) the initial contact (IC) of this sidestep, ACL injuries commonly occur (3, 11). At this time point, knee abduction moments (KAM) reach their maximum (12), which among other joint loads, such as internal rotation moments and anterior shear forces, are associated with ACL strain and therefore ACL injury risk (13, 14). Kinematics associated with higher multiplanar loads include an abducted and nearly extended knee, a flexed and abducted hip, ankle dorsiflexion (rearfoot landing) and trunk lean and rotation away from the movement direction (8, 15). These kinematic characteristics have been identified by video analyses of injury scenarios (3, 4, 16) and laboratory-based studies of knee loading (8), e.g., by examining COD maneuvers using three-dimensional motion capture analysis and force plates. While standardized, laboratory-based studies provide valuable information for understanding the biomechanical risk factors of ACL injuries, they may not fully capture the intricacies of real-world COD maneuvers and ACL injury situations. Therefore, the consequential next step driving COD injury research is to gain insight into more realistic movement patterns in ecologically valid settings. A recent opinion by Bolt et al. (17) highlights the importance of preserving athlete-environment relationship by e.g., including game-like experiments for performance and injury risk assessment. Currently, efforts are made to better recreate game-like conditions in COD assessments, which include (a) testing on the field, (b) adding attentional and anticipatory challenges to the tasks, (c) adding a performance focus and (d) moving away from isolated COD movements.

Firstly, a recent investigation demonstrated kinematic differences and higher movement variability in 45° unanticipated COD movements on the field compared to the laboratory (18) with the largest differences during the most crucial phase for ACL injury risk, the IC phase. These differences likely originate from standardization efforts in laboratory studies such as pre-defined movement paths, constrained foot placement, or controlled speeds, which result in limited room for players to use their preferred movement patterns (19). Further, altered environmental factors such as surface variations, e.g., turf vs. lab floor surfaces, can help explain kinematic differences. Recent advances in wearable motion capture systems based on inertial measurement units (IMU) have encouraged to further expand on-field assessments (20).

Secondly, many COD assessments have focused on preplanned movements where the COD direction is known before the run-up, while most natural CODs in game situations are unanticipated, reactive maneuvers (21). Studies including unanticipated CODs have identified the anticipation of movements, or lack thereof, as a determinant for injury risk, with evidence suggesting that the absence of motor pre-planning negatively impacts the COD movement towards riskier kinetics such as higher KAM and internal rotation moments (22, 23) and riskier kinematics such as reduced knee flexion or increased knee valgus (24). In unplanned tasks, altered kinematics are most likely a result of short preparation times (23). When responding to an external stimulus with short available response times, the use of motor control mechanisms for optimal motor planning (e.g., 25) is hindered and can result in movements with increased joint loads and reduced efficiency (21, 24, 26). The shorter preparation time is noticeable for example as later activation of the knee stabilizing musculature (27). A recent study by Gokeler et al. (28) revealed that non-contact ACL injuries often follow neurocognitive errors, highlighting the role of attentional, anticipatory and inhibitory demands in injury situations. Besides anticipatory challenges, attentional demands should therefore be increased in COD assessments (29), e.g., by introducing multiple COD angle options and directions when studying unplanned movements.

Thirdly, in game-scenarios a player's aim is usually focused on performance rather than injury prevention. Accordingly, COD movement assessments for injury risk factors should still demand maximum performance to mirror this focus on performance. The ability to perform CODs fast is an important part of athletic performance in soccer and other multidirectional sports, e.g., for creating space in offensive play or reacting to an opponent's action in defensive situations (30). This focus on speed creates a conflict between injury-safe movement execution and performance-enhancing movement execution (31). Performance-enhancing techniques, which shorten ground contact times and facilitate acceleration into a new movement direction, are often associated with higher knee loading, e.g., wide foot placement (hip abduction) and a stiffer knee landing (32). This discrepancy bears challenges for practitioners, coaches and athletes as to mitigate injury risk without deterring performance, highlighting the need for ACL research to provide guidelines for effective and adequate injury prevention programs (33).

Lastly, CODs rarely are isolated events in real sport settings but occur as part of multiple COD maneuvers, tackles and linear sprints within game flow (34, 35). Singling out COD movements might therefore alter timing, attention and lastly kinematics compared to real match scenarios. To tackle this limitation, more elaborate COD assessment tasks such as small sided games or football specific drills (18) have been tested. They were able to reveal different biomechanical risk patterns than in a comparable lab task. These efforts improve ecological validity of COD assessment but pose new challenges and limitations such as identifying CODs of interest and comparing the various COD movements of different angles and speeds. A sequential COD task could possibly bridge the gap between natural in-game movements and standardized testing. Therefore, the kinematic differences between a simple COD task (run up and cut) and a more extended COD assessment consisting of a sequence of multiple CODs on the field should be tested.

The purpose of this study is to address the above-mentioned shortcomings and investigate kinematic patterns and performance of COD maneuvers in a closer-to-reality setting including on-field testing with reduced constraints (e.g., for foot placement), multiple angles and directions in unplanned as well as preplanned conditions, and in a sequence of CODs. We hypothesized that (H1) kinematic characteristics at IC such as knee flexion, ankle dorsiflexion, hip abduction, trunk lateral lean, trunk rotation and pelvis rotation significantly differ between unplanned and preplanned CODs, with unplanned movements eliciting more injury-prone movements. Regarding performance, we hypothesized longer completion times in unplanned COD sequences compared to preplanned sequences. Additionally, we hypothesized that (H2) the kinematic patterns observed in unplanned scenarios further deteriorate when observing the last COD of an unplanned sequence compared to the first COD, potentially increasing injury risk.

## 2 Methods

### 2.1 Participants

Twenty healthy male participants (age: 23.8 ± 2.8 years; height: 180 ± 4.7 cm; weight: 73.8 ± 6.4 kg) took part in the study. They self-reported 16.7 ± 2.4 years of experience in multidirectional sports. All of them reported playing soccer as their main (*n* = 17) or secondary sports (*n* = 3), training 2.7 times per week on average. Some had additional COD experience from tennis and volleyball. Additionally, six participants regularly performed strength training. They reported no lower limb injuries within the last six months. Past but healed injuries included pulled hamstrings, ankle sprains, medial collateral ligament and meniscal injuries, but no ACL injuries. All participants were students at the University of Innsbruck and recruited from the University's recreational soccer teams. Players were not able to partake if they had recent injuries to the lower limbs, did not have prior experience in COD sports or if they were not enrolled as a student (due to insurance reasons).

Informed written consent was obtained after each participant received verbal and written information on the procedure. The study protocol was approved by the ethics committee of the University of Innsbruck (Board for Ethical Issues, Review Board Sport Science, ID 77/2022), following the principles of the Declaration of Helsinki.

An a-priori sample-size estimation with a significance level of 0.05, a desired power of 0.8, and an expected large effect size (*d* = 1.15) for the comparison of knee flexion angles of unplanned and preplanned CODs (36) resulted in a required sample size of nine participants. This estimation was performed using G*Power (v.3.1.9.7) (37) for *t*-tests analyzing the difference of two dependent means. A secondary estimation specific to linear mixed models [GLIMMPSE (38)] confirmed the required sample size for the statistical analysis used in the current study. To account for possible dropouts, the sample size was raised to ten and doubled to investigate the additional effect of COD sequence and angle, resulting in *n* = 20.

### 2.2 Experimental design

#### 2.2.1 Setup

**Figure 1:**
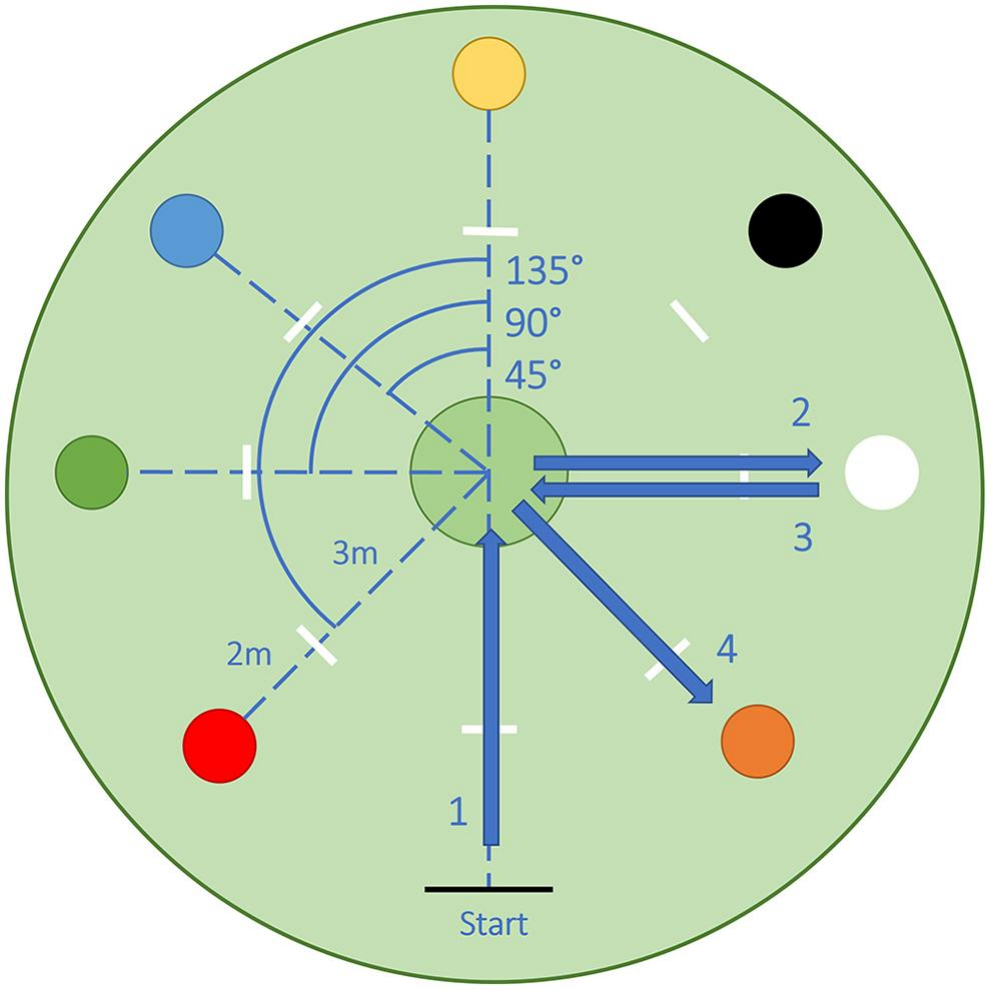
Experimental COD setup, colors represent target cones placed to create COD angles of 45°, 90° and 135° to the left and right to be performed in the center circle. Arrows and numbers indicate an exemplary COD sequence.

The study took place on an outdoor soccer field (50 × 25 m) in October of 2023. Participants performed maximum-speed COD maneuvers in a setup on artificial turf. A circular setup allowed for six COD options consisting of three COD angles of 45°, 90° and 135° to the left and right, respectively (Figure 1). Analogous to a clock, colored cones were placed at 8, 10, 12, 2 and 4 o'clock around the center. The starting line was placed at 6 o'clock. Participants performed a total of twelve sequences of COD maneuvers. A sequence consisted of a run up (5 m) and COD maneuver in the center circle directed at one of the target cones, followed by a 180° in a designated zone at the target cone back towards the center circle, where a second COD towards a new colored cone was performed. The trial ended when participants passed the second target cone after which they slowly came to a stop. Figure 1 depicts an exemplary running path for the color combination white and orange, resulting in a center circle COD of 90° to the right, a 180° cut at the white cone and another center circle COD of 135° COD to the left.

For preplanned COD sequences, participants were instructed on the subsequent movement directions by communicating the two colors of target cones before the run-up. In unplanned trials, participants received the information for the next target cone during the run up via an auditory cue at a 3 m mark from the center circle for the first and second center circle COD.

A trial was considered valid when participants performed a defined COD sidestep and touched the center circle (70 cm diameter) during both center CODs as well as a dedicated zone for the 180° COD, i.e., 40 cm in front of the target cone with one foot. If the participants clearly reacted wrongly (e.g., confusion of the colors) or did not touch within nor the lines of the target zones with at least one foot, the trial was repeated. Invalid preplanned trials were repeated. For invalid unplanned trials, the COD sequence was adapted to represent the same COD angles in different color combinations to maintain unpredictability.

#### 2.2.2 Protocol

All participants completed the study protocol (with a different set of COD sequences) one week in advance of the experiment to familiarize with the setup. A standardized warm-up including exercises 1–6 of the FIFA 11 + program (39) and various running drills and COD exercises of increasing intensity was performed prior to the COD test. Participants performed twelve COD sequences, of which the first and last COD of a sequence was analyzed, resulting in 24 investigated CODs for each participant. COD sequences were alternating between preplanned and unplanned trials and balanced to contain equal amounts of left and right directions. Additionally, trials were randomized within blocks to ensure everyone would receive the same COD angles and directions but in different order and expressed as different color combinations. Participants were instructed to perform each trial as fast as possible. They had 2 min of recovery time in between trials and were able to choose longer breaks if needed.

### 2.3 Measurements

#### 2.3.1 Performance

Light gates (Brower Timing Systems, Draper, UT, USA) with an accuracy of 0.001 s were placed at a height of 80 cm and one meter apart on the run up to measure approach speed 3 m before the first COD maneuver. Completion time of the COD trials was taken via a stopwatch from the start of the run-up until participants passed the second target cone of the sequence.

#### 2.3.2 Kinematics

To capture joint and segment kinematics, participants were equipped with eight IMU sensors (Ultium Motion) by Noraxon (Scottsdale, Arizona, USA). Sensors were placed on eight body parts: upper thoracic spine, back of the pelvis, both thighs, both shanks and the insteps of both shoes, following the respective manufacturer guidelines for sensor placement. A functional calibration including standing in the reference pose and walking back and forth in a straight line was performed for each subject. The IMU-model was scaled based on the height of the subject. Anatomical angles were calculated by the MyoMotion module of the system's corresponding Noraxon MyoResearch software (MR3 3.20.40; Scottsdale, Arizona, USA). The system uses a rigid-body skeletal model to convert the measured sensor frame orientations into segment orientations. Based on the segment orientation data, the software outputs anatomical angles as Cardan angles following the International Society of Biomechanics (ISB) recommendations for joint angle decomposition sequences. Pelvis orientation was based on the heading angle of the pelvis sensor in relation to the starting pose. For this analysis, joint angles of knee flexion, ankle dorsiflexion, hip abduction, trunk rotation and lateral lean were included as dependent variables, as well as pelvis rotation (orientation angle). The chosen variables of the lower limbs showed fair to good agreement compared to optical motion capture systems (20), however, no validation data is currently available for the pelvis and trunk motion. Although relevant to the ACL injury mechanism, variables of knee abduction and hip internal rotation were excluded from this analysis due to concerns about the validity of the IMU-based estimates (20, 40). In general, interpretations have to be made with possible accuracy shortcomings of IMU-based kinematics in mind.

Additionally, a camera [GoPro HERO9 (GoPro Inc., San Mateo, CA, USA)] recording at 50 Hz was placed ca. 4 m behind the starting line to provide video data of each trial, which was required for visual inspection and data segmentation.

#### 2.3.3 IC detection

For this analysis, kinematic characteristics were investigated at the IC of the COD sidestepping leg. The two investigated CODs were the ones performed in the center circle. First, the two CODs were identified visually by comparing the 2D video data and the Noraxon MyoMotion avatar reconstruction of the movement. A timeframe before to after the ground contact was selected manually for each COD respectively and served as a region of interest. A MATLAB script was used to find the IC automatically within the region of interest. The IC was defined as the peak of the resultant acceleration of the sidestepping leg's foot sensor (41). This IMU-based method for IC detection was validated in our motion laboratory using ground-embedded force plates (1,000 Hz, threshold 20 N) and revealed an average error of 0.013 s (*SD* = 0.004 s) based on 118 COD measurements. Although this error might be increased on softer surfaces such as artificial turf, the IMU-driven method based on the resultant accelerations' peak was accepted as a valid form of IC detection. The respective joint and orientation angles were extracted at the time of IC using a customized MATLAB script (R2023a, The MathWorks Inc., Natick, MA, USA).

### 2.4 Statistical analyses

All statistical procedures were completed in Jamovi 2.3 (42) using the GAMLj module for linear models (43). All dependent variables (six kinematic variables at IC and two performance variables) were investigated using linear mixed models with the random factor “participant”. Random effects included a participant-specific intercept to account for baseline differences between participants. Adding a random slope did not improve fit over the random-intercept-only model based on lower AIC/BIC values for the random-slope model and a non-significant likelihood ratio test (LRT) based on the log-likelihood (example for knee flexion: *p* = 0.068). Fixed-effect estimates and 95% confidence intervals were stable across supported random-effects structures. We therefore retained the random-intercept-only model for parsimony. The model used the fixed factors “condition” with four levels (first unplanned COD, first preplanned COD, last unplanned COD, last preplanned COD) and “COD angle” with three levels (45°, 90°, 135°) and investigated their interaction (condition × COD angle). Pelvis orientation data and run-up speed were only available for the first COD of a sequence. Therefore, a separate model was developed for those variables, which only regarded data of the first CODs and therefore used only two levels for the fixed factor “condition” (first unplanned, first preplanned) and the same three-level fixed factor for “COD angle”. Completion time was only available for the whole sequence. Therefore, the factor “COD angle” was adjusted to represent the combination of the two observed angles, which resulted in four possible angle sums: 90°, 135° 180° and 225°, while the factor “condition” included the levels unplanned and planned. Models were estimated using the restricted maximum likelihood (REML) method. We quantified effect sizes for fixed effects in our linear mixed models as partial *η^2^* computed from the F-statistics and their numerator and denominator degrees of freedom. This metric expresses the proportion of residual variance uniquely attributable to a given fixed effect after accounting for all other fixed effects and the specified random-effects structure. For interpretation, partial *η^2^* > 0.01 is considered a small effect, partial *η^2^* > 0.06 a medium effect and partial *η^2^* > 0.14 a large effect (44).

In the presence of significant interaction effects, *post-hoc* tests were conducted. Significant *post-hoc* tests were Bonferroni-corrected by multiplying the *p*-value by the amount of *post-hoc* comparisons relevant for the hypotheses tested. To test H1, nine *post-hoc* comparisons were regarded: comparisons of first preplanned vs. first unplanned CODs for every COD angle (three tests), comparisons between COD angles within the first unplanned COD trials (three tests) and within the first preplanned trials (three tests). For H2, the following nine *post-hoc* tests were of importance: comparisons of the first to last COD of the unplanned trials for every COD angle (three tests), comparisons between COD angles within the first unplanned trials (three tests) and within the last unplanned trials (three tests). The significance level for all statistical analyses was set to alpha = 0.05. Descriptive statistics are expressed as mean (M) and standard error (SE). Missing data was simply excluded; no imputation was performed. The linear mixed-effects model accommodates unequal numbers of observations per participant, so unbalanced data were permissible. Due to occasional foot sensor problems, 15 out of 480 ankle angles had to be excluded.

## 3 Results

### 3.1 Performance variables

**Figure 2:**
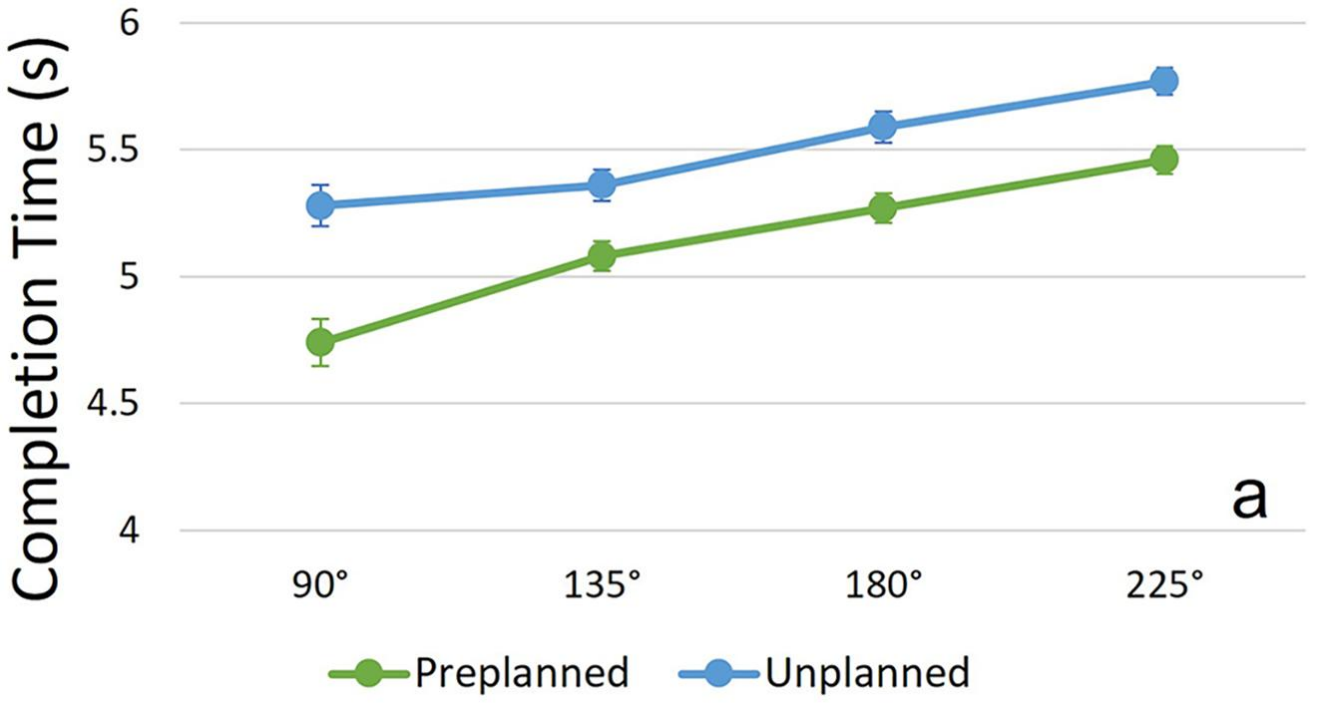
Completion time (*y*-axis) of whole COD sequences, presented for the sum of COD angles performed in one sequence (*x*-axis), both for unplanned and preplanned trials. Circles and error bars show the estimated marginal means and standard errors for each combination of condition and COD angle. “a” indicates a sign. main effect of anticipation condition.

A significant main effect for condition [*F*(1,215) = 113.05, *p* < 0.001, partial *η^2^* = 0.345] as well as for COD angle [*F*(3,215) = 67.02, *p* < 0.001, partial *η^2^* = 0.483] was found for completion time (Figure 2). Participants performed unplanned COD sequences (*M* = 5.5 s, *SE* = 0.05 s) significantly slower than preplanned sequences (*M* = 5.14 s, *SE* = 0.05 s). Completion times significantly increased with sharper COD angle combinations. No significant interaction effect (condition × COD angle) was found for completion time [*F*(3,218) = 1.48, *p* = 0.222, partial *η^2^* = 0.02].

There were no significant effects of condition or COD angle on run-up speed (condition: *F*(1,215) = 0.719, *p* = 0.397, partial *η^2^* = 0.003, COD angle: *F*(2,216) = 0.234, *p* = 0.791, partial *η^2^* = 0.002, condition × COD angle: *F*(2,216) = 2.190, *p* = 0.114, partial *η^2^* = 0.02). The mean run-up speed was *M* = 3.74 m/s (*SE* = 0.107 m/s) in unplanned conditions and *M* = 3.81 m/s (*SE* = 0.107 m/s) in preplanned conditions.

### 3.2 Kinematic variables

#### 3.2.1 Main and interaction effects

A significant main effect for condition was observed regarding knee flexion angle [*F*(3,449) = 13.97, *p* < 0.001, partial *η^2^* = 0.085], trunk rotation [*F*(3,449] = 5.27, *p* < 0.001, partial *η^2^* = 0.034 and pelvis orientation [*F*(1,215) = 19.881, *p* < 0.001, partial *η^2^* = 0.085]. No statistically significant main effects for condition were found for ankle dorsiflexion [*F*(3,434) = 0.702, *p* = 0.551, partial *η^2^* = 0.005], hip abduction [*F*(3,449) = 2.23, *p* = 0.083, partial *η^2^* = 0.015] or trunk lateral lean [*F*(3,449) = 1.76, *p* = 0.155, partial *η^2^* = 0.012].

A significant interaction effect (condition × COD angle) was found for knee flexion [*F*(6,450) = 5.08, *p* < 0.001, partial *η^2^* = 0.063], trunk rotation [*F*(6,451) = 2.84, *p* = 0.010, partial *η^2^* = 0.036] and hip abduction [*F*(6,451) = 2.60, *p* = 0.017, partial *η^2^* = 0.033]. Interaction effects of ankle dorsiflexion [*F*(6,436) = 1.208, *p* = 0.301], trunk lateral lean [*F*(6,451) = 1.39, *p* = 0.218, partial *η^2^* = 0.018] and pelvis orientation [*F*(2,222) = 0.435, *p* = 0.648, partial *η^2^* = 0.004] were not significant.

A significant main effect for the COD angle (45°, 90°, 135°) was observed for all joint angles with regard to H1 and H2 (knee flexion: *F*(2,449) = 48.82, *p* < 0.001, partial *η^2^* = 0.179; ankle dorsiflexion: *F*(2,434) = 47.427, *p* < 0.001, partial *η^2^* = 0.179; hip abduction: *F*(2,449) = 152.36, *p* < 0.001, partial *η^2^* = 0.404; trunk rotation: *F*(2,449) = 32.27, *p* < 0.001, partial *η^2^* = 0.126; trunk lateral lean *F*(2,449) = 8.87, *p* < 0.001, partial *η^2^* = 0.038; pelvis orientation (only H1): *F*(2,222) = 136.966, *p* < 0.001, partial *η^2^* = 0.552). Means and standard errors for all kinematic variables can be found in Table 1.

**Table 1:** Means and standard errors for kinematic variables, split by condition and first/last COD.

Kinematic variable	Preplanned															
	First COD								Last COD							
	**45°**		**90°**		**135°**		**Overall**		**45°**		**90°**		**135°**		**Overall**	
	**M**	**SE**	**M**	**SE**	**M**	**SE**	**M**	**SE**	**M**	**SE**	**M**	**SE**	**M**	**SE**	**M**	**SE**
Knee Flexion	38.8	1.73	35.9	1.67	36.0	1.8	36.9	1.43	41.0	1.73	32.3	1.80	31.4	1.67	34.9	1.43
Ankle Dorsiflexion	3.08	1.79	−2.58	1.71	−3.6	1.91	−1.03	1.35	6.53	1.82	−1.99	1.89	−2.43	1.71	0.71	1.35
Hip Abduction	1.41	1.35	13.89	1.3	13.31	1.42	9.54	1.04	1.01	1.35	13.34	1.42	16.22	1.3	10.19	1.04
Trunk Rotation	3.07	1.63	4.19	1.56	7.63	1.72	4.96	1.23	6.09	1.63	6.79	1.72	9.20	1.56	7.36	1.23
Trunk Lateral Lean	18.2	1.48	15.8	1.42	13.3	1.56	15.8	1.15	16.6	1.48	17.8	1.56	15.0	1.42	16.5	1.15
Pelvis Orientation	11.81	2.53	35.92	2.37	50.61	2.74	32.8	1.55								
	Unplanned															
	First COD								Last COD							
	**45°**		**90°**		**135°**		**Overall**		**45°**		**90°**		**135°**		**Overall**	
	**M**	**SE**	**M**	**SE**	**M**	**SE**	**M**	**SE**	**M**	**SE**	**M**	**SE**	**M**	**SE**	**M**	**SE**
Knee Flexion	43.8	1.76	41.3	1.82	38.0	1.65	41.1	1.43	45.1	1.7	32.6	1.67	32.5	1.87	36.7	1.43
Ankle Dorsiflexion	5.81	1.88	−2.37	1.92	−3.55	1.68	−0.04	1.35	7.41	1.83	−0.93	1.74	−6.82	2.01	−0.12	1.37
Hip Abduction	3.14	1.39	10.57	1.44	11.06	1.27	8.26	1.05	0.65	1.32	12.49	1.29	12.15	1.49	8.43	1.05
Trunk Rotation	0.08	1.68	3.68	1.75	12.33	1.52	5.36	1.23	4.59	1.59	7.48	1.55	14.21	1.81	8.76	1.23
Trunk Lateral Lean	16.1	1.52	15.9	1.58	14.9	1.39	15.6	1.16	18.3	1.45	20.1	1.42	14.2	1.63	17.6	1.16
Pelvis Orientation	1.05	2.63	25.9	2.79	44.17	2.29	23.7	1.56								

#### 3.2.2 H1—preplanned vs. unplanned CODs

At the IC of unplanned first COD movements, participants showed higher knee flexion angles (*M* = 41.1°, *SE* = 1.43°) and less pelvis orientation towards the new movement direction (*M* = 23.7°, *SE* = 1.56°) compared to preplanned first CODs (knee flexion: *M* = 36.9°, *SE* = 1.43°, *p* < 0.001; pelvis orientation: *M* = 32.8°, *SE* = 1.55°).

**Figure 3:**
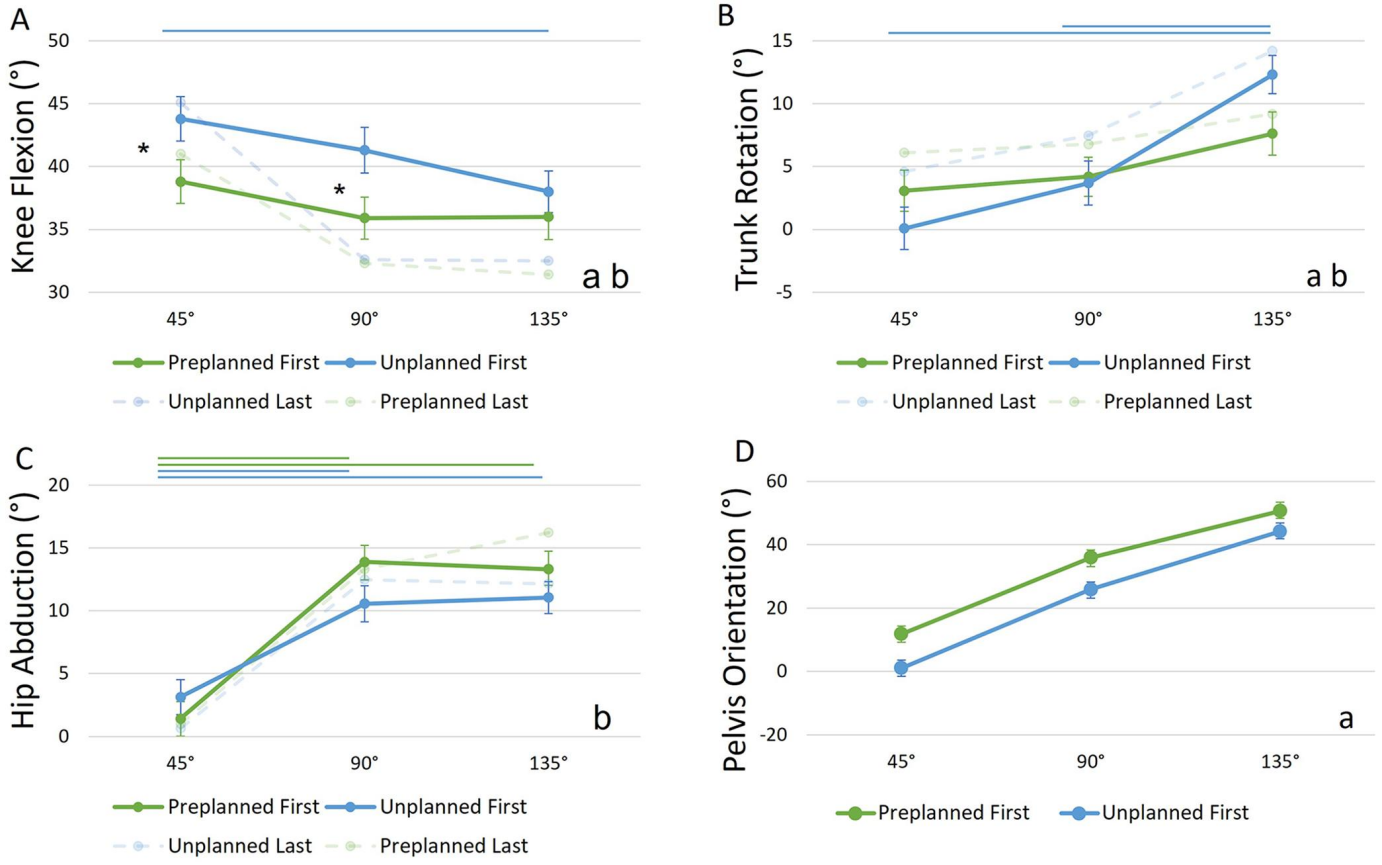
Joint **(A–C)** and orientation angles **(D)** (*y*-axis) at different COD angles (*x*-axis) for different anticipation conditions and sequence numbers. Circles and error bars show the estimated marginal means and standard errors for each combination of condition and COD angle. Data for H1 is highlighted, while data for H2 is faded. “a” indicates a sign. main effect of condition, “b” indicates a sign. interaction effect (condition × COD angle). Asterisks indicate sign. *post-hoc* tests between “Preplanned First” and “Unplanned First” and straight lines indicate sign. *post-hoc* tests between COD angles (green for “Preplanned” and blue for “Unplanned”).

For knee flexion, *post-hoc* testing revealed significant higher knee flexion angles in unplanned first CODs between 45° COD angles (*p* = 0.027, *M* = 43.8°, *SE* = 1.76°) as well as 90° COD angles (*p* = 0.018, *M* = 41.3°, *SE* = 1.82°) compared to preplanned scenarios (45°: *M* = 38.8°, *SE* = 1.73°; 90°: *M* = 35.9°, *SE* = 1.67°). Additionally, knee flexion was significant lower in sharp (135°) COD angles (*M* = 38°, *SE* = 1.65°) compared to 45° angles (*M* = 43.8°, *SE* = 1.76°) of unplanned first CODs (*p* = 0.009) but not for preplanned first CODs. *post hoc* testing for trunk rotation showed a significant increase in trunk rotation from 45° (*M* = 0.08°, *SE* = 1.68°) to 135° (*M* = 12.33°, *SE* = 1.52°, *p* = 0.009) as well as from 90° (*M* = 3.68°, *SE* = 1.75°) to 135° (*p* = 0.009) in the unplanned but not in the preplanned condition. *post hoc* testing identified a significant increase in hip abduction in both conditions from 45° (unplanned: *M* = 3.14°, *SE* = 1.39°; preplanned: *M* = 1.41°, *SE* = 1.35°) to 90° (unplanned: *M* = 10.57°, *SE* = 1.44°, *p* = 0.009; preplanned: *M* = 13.89°, *SE* = 1.3°, *p* = 0.009) and from 45° to 135° (unplanned: *M* = 11.06°, *SE* = 1.27°, *p* = 0.009; preplanned: *M* = 13.31°, *SE* = 1.42°, *p* = 0.009) with a larger increase in the preplanned scenario (Figure 3).

#### 3.2.3 H2—first vs. last unplanned CODs

Knee flexion was significantly lower in the last (*M* = 36.7°, *SE* = 1.43°) compared to first CODs (*M* = 41.1°, *SE* = 1.43°, *p* < 0.001) and trunk rotation was found to be increased in last (*M* = 8.76°, *SE* = 1.23°) compared to first COD of a sequence (*M* = 5.36°, *SE* = 1.23°, *p* = 0.002). Pelvis orientation was not examined for the last CODs.

**Figure 4:**
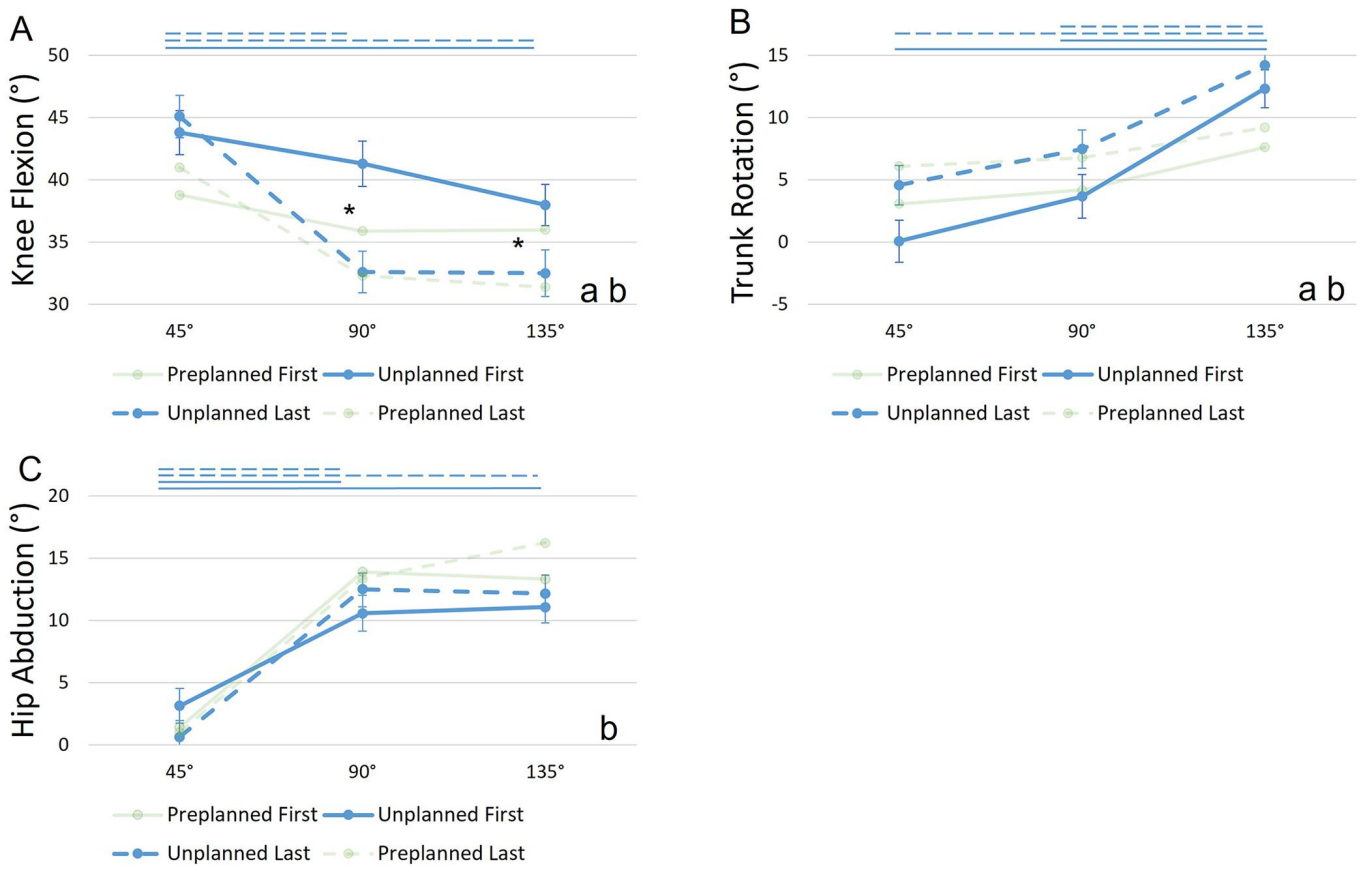
Joint angles **(A–C)** (*y*-axis) at different COD angles (*x*-axis) for different anticipation conditions and sequence numbers. Circles and error bars show the estimated marginal means and standard errors for each combination of condition and COD angle. Data for H2 is highlighted, while data for H1 is faded. “a” indicates a sign. main effect of condition, “b” indicates a sign. interaction effect (condition × COD angle). Asterisks indicate sign. *post-hoc* tests between “Unplanned First” and “Unplanned Last” and straight lines indicate sign. *post-hoc* tests between COD angles (solid for “First” and dashed for “Last”).

Post hoc test showed a significantly lower knee flexion angle in the last COD compared to the first COD of unplanned sequences in both the 90° (first: *M* = 41.3°, *SE* = 1.82°; last: *M* = 32.6°, *SE* = 1.67°, *p* = 0.009) and 135° angle (first: *M* = 38°, *SE* = 1.65°; last: *M* = 32.5°, *SE* = 1.87°, *p* = 0.018). Additionally, in unplanned first CODs knee flexion angles differed significantly between 45° (*M* = 43.8°, *SE* = 1.76°) and 135° (*p* = 0.009). Knee flexion in unplanned last CODs differed significantly between 45° (*M* = 45.1°, *SE* = 1.7°) and 135° (*p* = 0.009) as well as between 45° and 90° angles. Trunk rotation significantly increased from 45° (first: *M* = 0.08°, *SE* = 1.68°; last: *M* = 4.59°, *SE* = 1.59°) to 90 (first: *M* = 3.68°, *SE* = 1.75°; last: *M* = 7.48°, *SE* = 1.55°) as well as from 90° to 135° (first: *M* = 12.32°, *SE* = 1.52°; last: *M* = 14.21°, *SE* = 1.81°) in both first (*p* = 0.009) and last CODs (*p* = 0.009). Hip abduction significantly increased from 45° (first: *M* = 3.14°, *SE* = 1.39°; last: *M* = 0.65°, *SE* = 1.32°) to 90° (first: *M* = 10.57°, *SE* = 1.44°; last: *M* = 12.49°, *SE* = 1.29°) as well as from 45° to 135° (first: *M* = 11.06°, *SE* = 1.27°; last: *M* = 12.15°, *SE* = 1.49°) in both first (*p* = 0.009) and last CODs (*p* = 0.009, Figure 4).

## 4 Discussion

This study investigated kinematic patterns of COD movements in unplanned and preplanned conditions as well as differences in the first and last CODs of a sequence of unplanned movements. Our hypothesis on anticipation effects (H1) was only partially supported, as both injury-provoking as well as risk-mitigating movement adaptations were found in four of the measured joint angles at IC (knee flexion, hip abduction, trunk rotation and pelvis orientation) in the unplanned condition. In support of H2, injury risk-provoking movements were more pronounced in unplanned trials at the end of a COD sequence compared to the first COD, noticeable in biomechanical adaptations in knee flexion and trunk rotation angles associated with higher injury risk. Due to the performance focus of the task and the reduced COD completion times in unplanned trials, the kinematic findings will be discussed both from an injury risk perspective as well as from a performance perspective.

### 4.1 COD kinematics during preplanned vs. unplanned trials—a performance-injury conflict

Kinematic patterns related to ACL injury risk could not solely be attributed to one of the anticipation conditions. Rather, the movement patterns found in preplanned CODs show both risk-mitigating as well as injury-provoking mechanisms. While trunk and pelvis show “safer” transversal motion in line with our hypothesis, the biomechanical patterns of the wider hip angle in the frontal plane and the more extended knee in the sagittal plane during preplanned scenarios are more in line with common ACL injury mechanisms, contradicting our hypothesis. In contrast, all kinematic characteristics observed in the preplanned condition can be related to COD techniques enhancing performance. These findings can be explained in the context of the performance-injury conflict (31, 32). Participants performed preplanned COD sequences faster than unplanned COD sequences. To exclude the effect of differences in the run up on completion time or kinematics at IC (45), run-up speed was included in the analysis and did not differ systematically between the conditions. Therefore, the performance of the COD sidestep itself and subsequent reacceleration must be enhanced in preplanned movements compared to unplanned movements. We allocate this performance advantage to appropriate movement planning, such as earlier trunk rotation and pelvis orientation.

A pelvis rotation towards the new intended movement direction, as seen in the preplanned condition, is considered an efficient strategy for both COD performance as well as injury prevention by initiating whole-body rotation before ground contact and therefore reducing rotational demands for the lower limbs during the stance phase (46). Due to reduced preparation, this technique for reducing risk and enhancing performance was not achievable in unplanned CODs. Additionally, in unplanned CODs at angles exceeding 90° we observed a more pronounced increase in trunk rotation away from the movement direction. This misalignment is linked to higher injury risk (32), possibly by increasing internal rotation moments in the knee (47) contributing to ACL loading (13). This highlights the demands for trunk stabilization at sharper COD angles. From a performance view, proper trunk rotation towards the new movement direction improves cutting speed (48). Both adequate pelvis and trunk positioning were lacking in unplanned scenarios, resulting in higher-risk biomechanical patterns in support of our first hypothesis.

Participants employed other movement patterns enhancing performance at the potential cost of higher knee loads (less knee flexion, more hip abduction) in preplanned CODs. On the other hand, unplanned scenarios showed less “risky” angles in hip and knee joints. Participants landed with lower hip abduction angles in unplanned scenarios, while there was a stronger increase in hip abduction in preplanned conditions from 45° to sharper angles. High hip abduction and the resulting wide foot placement is considered to increase COD performance by reducing contact times and facilitating acceleration into the new movement direction (32). However, it is also closely related to higher KAM, by placing the center of mass (COM) further from the center of pressure (COP), increasing injury risk (47). A reduction of hip abduction by employing a narrower foot placement is considered a technique to lower ACL injury risk (8). As a caveat, we would like to mention, that, while the validity of IMU-derived hip abduction has been found to be variable across participants (20), we still included hip abduction as an outcome variable in the interest of gaining insight into the lower-body frontal plane in ecologically valid testing.

Additionally, a more extended knee was observed in the preplanned scenarios, which benefits performance by reducing contact times and generating lateral forces for a push off into the new direction (32, 49). The extent of the sagittal knee movements' contribution to the ACL injury mechanism has been debated (21, 50) but there is evidence that a nearly extended knee (0–40°; 22) and a stiff knee landing increases injury risk by facilitating anterior tibial translation contributing to ACL load (51, 52). A bent knee, on the other hand, can facilitate the hamstrings to actively prevent anterior tibial translation (53). In fact, a “soft landing” using a higher knee flexion range of motion (ROM) to absorb and dissipate ground reaction forces is a common technique instruction to reduce injury risk (54). This technique, however, comes at the cost of performance and, therefore, is unappealing for coaches and athletes (32). In unplanned scenarios with less preparation time, participants exhibited greater knee flexion. This finding was consistent with earlier research. With some exceptions (55), the majority of previous studies have reported higher knee flexion angles in tasks with reduced preparation times (21, 56), even though unplanned tasks are generally considered riskier and produced higher KAM (23). The flexed knee likely reflects an effort of athletes to lower their COM in response to the task's unpredictability similar to what has been observed when individuals run on unstable surfaces (57). So, while the lacking trunk and pelvis positioning might be a sign of missing preparation time, the lower knee flexion and hip abduction angle could be regarded as means of readiness and robust motor control strategy in uncertain situations, preparing for the unknown.

It should be noted, that, with an average approach speed of 3.74 m/s in the unplanned condition and a cue 3 m before the COD target, participants had about 800 ms to react to the cue. Other studies investigating unplanned COD movements used shorter available time to react (ATR). While shorter times such as 300 ms are recommended to reveal unanticipated responses, anticipation effects were found in conditions with ATR up to 600 ms (24, 58). In contrast to the classic left vs. right COD protocols for one given COD angle, our protocol included six COD options (2 directions×3 angles), which increases the cognitive demand for preparing a movement response to the directional cue (24). Further, we observed that for our protocol shorter ATRs often lead to undesired compensatory movements delaying the actual response, such as small quick steps instead of a sidestep. Therefore, our ATR was chosen specifically for our COD protocol and the skill level of our participants, who were recreational soccer players. Since our task still revealed kinematic differences between the anticipation conditions, we conclude that the chosen ATR was appropriate but we aim to include shorter cue times for comparison in following studies.

### 4.2 More injury-prone COD kinematics towards the end of a COD sequence

Although protective measures and a robust control strategy seem appropriate for uncertain scenarios, such strategies are typically not observed in realistic game situations and realistic injury scenarios, which report e.g., low knee flexion angles just before ACL injuries (3). This discrepancy could come from divergent COD kinematics between isolated CODs, which are typically investigated in biomechanical studies, and CODs within a movement sequence, representative of real-world scenarios. To employ a more game-like situation, where CODs follow other cuts or sprints, we incorporated a sequence of CODs. Results showed that protective measures seen in unplanned isolated CODs were not found for CODs at the end of a sequence of unplanned movements. While an isolated unplanned COD resulted in some injury-protective kinematics (H1), CODs later in a sequence of movements showed increased ACL injury risk patterns.

A simple sequential COD task was able to provoke and expose potentially risky movement patterns in unplanned movements. In the last COD, participants touched down with a more extended knee at COD angles sharper than 90°, which is considered to increase ACL injury risk (as explained above; 52). Similarly, they rotated their trunk further away from the intended new movement direction in the last COD in unplanned sequences and showed an increase in hip abduction at sharper COD angles, again potentially increasing injury risk (as explained above; 32). These high-risk kinematic characteristics, e.g., the low knee flexion angle, were comparable to angles observed in isolated preplanned COD scenarios. Therefore, performing multiple CODs in a row seems to “overwrite” the protective strategy observed in isolated unplanned scenarios. While in preplanned movements, higher knee loads through kinematics such as an extended knee might be tolerable due to appropriate motor planning and adequate muscle activation, these movements might be potentially harmful, when they occur in an unplanned scenario. Future research should be focused on comparing knee joint loading and muscle activation between isolated and sequential CODs to investigate these assumptions.

The adaptation between first and last COD are likely due to increased cognitive and physical demands of the last COD, including spatial reorientation within the six-option COD course and reacceleration demands. A straight run up, as before the first COD, allowed participants to focus and prepare for the uncertainty of the first task and (unconsciously) select movement strategies balancing load and injury risk. However, the spatial reorientation and reacceleration demands before the last COD may have occupied the sensorimotor system enough to affect motor planning, resulting in higher-risk movements. It is known that cognitive and attentional demands influence decision making and motor planning capabilities. And as task complexity and cognitive demands increase, movement planning further deteriorates (24). Therefore, the added cognitive demand and further limited preparation time due to the preceding high-intensity 180° cut might have influenced movement planning and execution negatively. Similarly, secondary tasks during cutting (e.g., counting backwards) divide focus and attention and have been shown to deteriorate movement execution (24, 59). And while little is known about the demands of sequential CODs, COD research including more complex COD tasks or small sided games have shown a similar effect (18). CODs in game situations, and hence in injury situations, often occur as part of a chain of movements (linear sprints, acceleration/deceleration, stops, multiple CODs; 34) with elevated cognitive demands. Additionally, ACL injuries often occur following neurocognitive errors (28) and links were found between low neurocognitive functions and ACL injuries (60, 61). Training of neurocognitive functions, playing a critical part in motor control, could therefore have benefits for knee loading and injury prevention (29). COD assessments including cognitive and attentional challenges are a first step into understanding this relationship. By recreating real-life situations, we get a better insight into the circumstances, which contribute to the occurrence of high-risk movements and high knee loading.

One factor to consider when comparing the first and last COD of a sequence is a potential difference in run up speed (45), which was not measured nor controlled before the last COD. While the first COD is following a linear sprint from a starting position, the last COD is preceded by a 180° COD. If anything, we assume participants approached the last COD slower due to the preceding de- and reacceleration phase of the 180° COD. Prior work indicates that slower approaches generally yield COD movement characteristics associated with lower ACL injury risk (62). Thus, a speed difference alone would be expected to bias the last COD toward “safer” COD movements. In contrast, our findings indicate injury-provoking kinematics at the last COD, suggesting that we measured an effect of sequential-task demands rather than an effect of approach speed. We acknowledge the absence of last-COD speed measurements as a limitation for our interpretation. Where possible, future studies should estimate approach speed (e.g., from IMUs using pelvis velocity as a proxy) and/or include approach speed as a covariate in the analyses.

The findings encourage the use of more complex COD tasks for realistic assessments of movement strategies. A sequence of unplanned CODs seems more suitable to provoke injury-prone COD kinematics, which can then be the basis for risk screening or intervention assessments under more realistic conditions. As a note of caution, we would like to stress that all COD assessments must carefully balance the sensitivity of the protocol for exposing potentially harmful movement patterns with the actual risk of injury during the assessment. Given our thorough warm-up routine and the absence of opponents or balls, we were confident to not harm our participants.

As a side note, a main effect of the COD angle (across all anticipation conditions) was significant for all observed joint angles. This highlights the different demands when performing CODs of different angles, resulting in distinct biomechanical patterns. This angle-dependency is congruent with earlier COD research (63). Therefore, results of CODs assessments can only be adequately compared within the same COD angles. Additionally, when investigating ACL injury mechanisms in COD movements, it can be important to examine multiple angles, as specific risk-increasing characteristics (such as trunk rotation) seem to be most prominent for certain angles (e.g., in angles sharper than 90°).

We recognize a few limitations in our study, such as shifting weather and field conditions, along with the lack of kinetic data. In future research, we plan to incorporate estimates of joint moments and loading. In regard to our outcome variables, we mention that IMU-derived kinematics may be affected by sensor drift, soft-tissue artifact, and calibration/alignment errors, which can reduce accuracy but were kept to a minimum by following the system manufacturer's guidelines. While we understand the challenges of replicating real-life sports situations, we also see the value in doing so. Despite these obstacles, our method successfully highlighted how athletes adjust their movements during unexpected CODs with various angle choices, all within a realistic on-field environment. The findings of this study are currently limited to male athletes. Due to high incidences of ACL injuries in female soccer players (64), replicating the protocol with a female population could give insight into sex-specific biomechanics and motor control of CODs.

In conclusion, this study highlights how both task anticipation and the sequence position within a series of COD maneuvers significantly influence lower limb, pelvis and trunk kinematics, with direct implications for ACL injury risk. These results support the idea that performance and injury risk often exist in conflict, with athletes intuitively prioritizing performance-enhancing kinematics in preplanned conditions over injury risk-mitigating movement strategies. Unplanned CODs induced a mix of protective and risk-associated movement adaptations, characterized by safer sagittal plane knee and frontal plane hip kinematics but less favorable trunk and pelvis alignment. However, these protective mechanisms were not found when observing the last COD of a sequence of unplanned CODs. Distinct kinematic differences between the first and last COD maneuver of an unplanned sequence revealed more risk-associated kinematic characteristics in the final COD movement of an unplanned sequence, marked by a more extended knee, higher hip abduction in sharper angles and misaligned trunk rotation. This suggests combined cognitive and physical load as a contributing factor to movement deterioration and highlights the need for prevention programs addressing both factors: Besides strengthening and technique exercises that also maintain performance, training programs should include reactive tasks which train quick decision-making, selective attention, and spatial orientation skills to facilitate motor planning in cognitively demanding situations. Collectively, these findings emphasize the role of limited preparation time and increased neurocognitive demands in constraining motor planning during COD movements, leading to altered biomechanical patterns that may increase the risk for ACL injuries.

## Data Availability

The raw data supporting the conclusions of this article will be made available by the authors, without undue reservation.
